# Ocular squamous cell carcinoma (OSCC) in a female buffalo

**DOI:** 10.17221/26/2025-VETMED

**Published:** 2026-02-27

**Authors:** Rinaldo Batista Viana, Adryele Araujo Borges Lima, Joao Marcelo de Sousa Soares, Giovanna Meireles Borges, Dyogines Araujo Marques, Sacha Manuelly da Silva Lobato, Deyvid de Menezes Melo, Jamyle Carollyne Melem Santos, Liane do Socorro Bremgarter, Pedro Eduardo Zezema, Suellen da Gama Barbosa Monger, Gabriela Melo Alves dos Santos, Pedro Paulo Maia Teixeira, Gilvando Rodrigues Galvao, Jose Dantas Ribeiro Filho, Bruno Moura Monteiro

**Affiliations:** ^1^Federal Rural University of the Amazon, Belém, PA, Brazil; ^2^Federal Institute of Education, Science and Technology of Amazonas, Manaus, AM, Brazil; ^3^Evandro Chagas Institute, Belém, PA, Brazil; ^4^Federal University of Pará, Castanhal, PA, Brazil; ^5^Federal University of Viçosa, Viçosa, MG, Brazil

**Keywords:** Amazonia, buffaloes, eye, ocular neoplasia

## Abstract

Ocular squamous cell carcinoma (OSCC) is an epithelial neoplasm that affects the ocular and periocular tissues, often associated with factors such as exposure to ultraviolet radiation. The disease is rarely reported in buffalo, particularly regarding its progression and treatment. This report describes a case of a buffalo with a pink mass in the right eye showing signs of inflammation. After clinical examination and initial treatment with topical solutions and systemic drugs, the tumour continued to grow. As a result, surgery was performed to remove the mass while preserving the eyeball and third eyelid. The procedure was successful, and histopathological analysis confirmed the diagnosis of OSCC. Postoperative recovery was satisfactory. It was concluded that early surgical treatment followed by medical treatment allowed complete recovery in the buffalo with OSCC.

Ocular squamous cell carcinoma (OSCC) is a primary neoplasm of epithelial origin that mainly affects ocular and periocular tissues (Carvalho et al. 2005) and is characterised by the differentiation of keratinocytes (Costa et al. 2019). This disease is prevalent in mucocutaneous junctions, including the palpebral conjunctiva, nictitating membrane, and cornea (Carvalho et al. 2005; Krishna et al. 2020). The OSCC tends to be more locally invasive than it is to metastaside (Santos and Alessi 2016). However, dissemination of cancer cells to regional lymph nodes or the lungs may occur (Srivastav et al. 2022).

Environmental factors, such as prolonged exposure to ultraviolet radiation, account for the majority of OSCC cases (Dubielzig 2002). Therefore, the incidence of this disease in cattle is more frequent in Hereford and Simmental herds, due to the lower degree of periocular and corneoscleral pigmentation (Anderson and Badzioch 1991). Even so, reports of OSCC in animals with black skin, such as zebu cattle (Soares et al. 2023) and buffaloes (Islam et al. 2017), have been documented.

In Brazil, prevalence rates of OSCC in cattle are 4.0% (Pires 2018), 14.4% (Tessele and Barros 2016) and 18.2% (Reis et al. 2017). However, only one study of OSCC in buffaloes was found (Oliveira 2020), which described two cases. This type of neoplasia is rarely reported in buffaloes, especially regarding OSCC progression and treatment. This can be explained by the option of enucleating the affected eyes (Krishna et al. 2020) or slaughtering the sick animal.

Despite reports, there is a paucity of observational data on a minimally invasive treatment that preserves the eyeball. The objective of this paper is to present a case study of an Amazonian buffalo affected by OSCC, detailing early diagnosis, tumour progression, surgical treatment, and subsequent recovery.

## Case description

On January 25, 2024, a Murrah-Mediterranean crossbreed aged eight years was treated at the Biotério Unidade de Bubalinocultura Leiteira Eva Daher Abufaiad (BUBali) at the Federal Rural University of the Amazon (Ufra).

The animal was found to have a pink mass and mucoid secretion in the right eye, accompanied by an inflammatory response. On ophthalmological examination (D-28), the left eye revealed epiphora, blepharospasm, and hyperaemia of the palpebral conjunctiva. Heart rate, respiratory rate, and rectal temperature were monitored daily until the animal was discharged.

**Figure 1:**
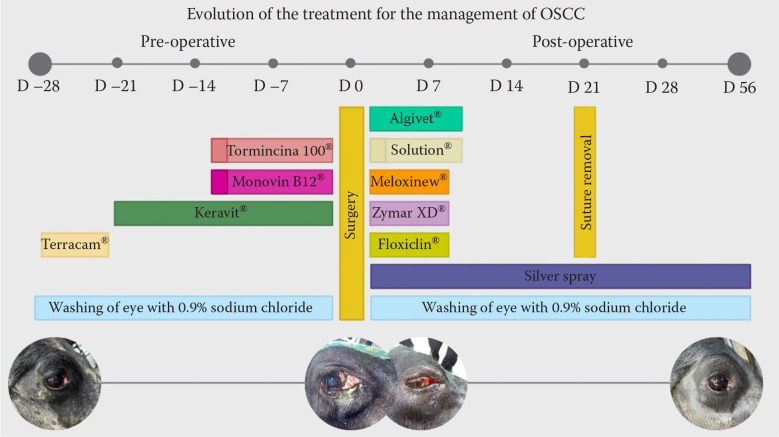
Evolution of the treatment for the management of squamous cell carcinoma (OSCC)

The initial treatmetnt involved ocular lavage with 0.9% sodium chloride and the application of an antimicrobial and anti-inflammatory aerosol solution (Terramicina^®^; Agener União Saúde Animal Ltda, São Paulo, Brazil) (Figure 1). After seven days (D-21), with no significant improvement and a progressive increase in the mass, an ophthalmic ointment containing gentamicin, hydrocortisone, and vitamins A and D (Keravit^®^; Vetnil Ltda, São Paulo, Brazil) was introduced. In the second week (D-14), a single dose of vitamin B12 (Monovin B12^®^; Bravet Ltda, Rio de Janeiro, Brazil) and oxytetracycline (Tormicina 100^®^; Fabiane Saúde Animal Ltda) was administered.

**Figure 2:**
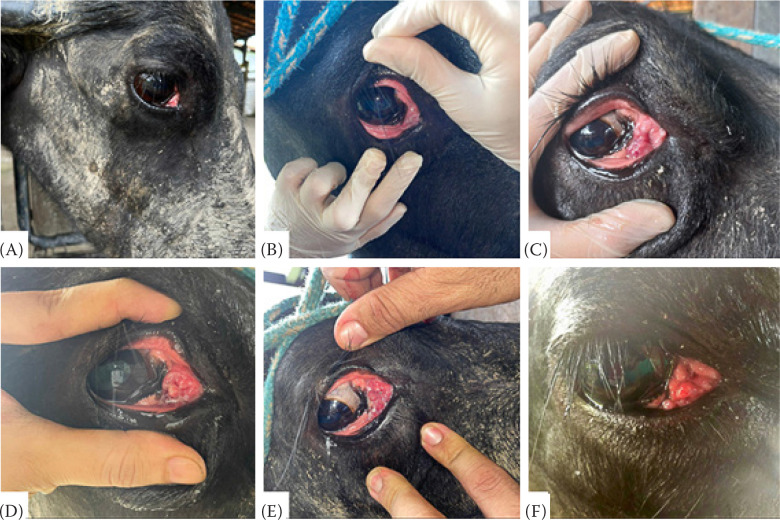
Preoperative evolution in four weeks of squamous cell carcinoma in the right eye of a female buffalo (A) Day –28; (B) day –21; (C) day –14; (D,E) day –7; (F) day 0

After 28 days of symptomatic treatment, the tumour became evident (Figure 2), and surgical excision of the neoplastic mass was decided. Preoperative care included a 24-hour food fast (D-1) and a six-hour water fast, with continuous monitoring until the day of surgery (D0). The excision technique aimed to preserve the eyeball and the third eyelid.

For the anaesthetic protocol, 0.01 mg/kg of 2% xylazine (Xilazin^®^; Syntec Ltda, São Paulo, Brazil) was administered intravenously for chemical restraint. Physical restraint was achieved by placing the animal in right lateral recumbency, with its head secured over the left flank, followed by antisepsis of the area. Local anaesthesia was performed with 2% lidocaine (Lidovet^®^; Bravet Ltda, Rio de Janeiro, Brazil), including blocks of the supraorbital, lacrimal, intraocular, and zygomatic nerves, as well as infiltrative anaesthesia of the upper and lower eyelids.

**Figure 3:**
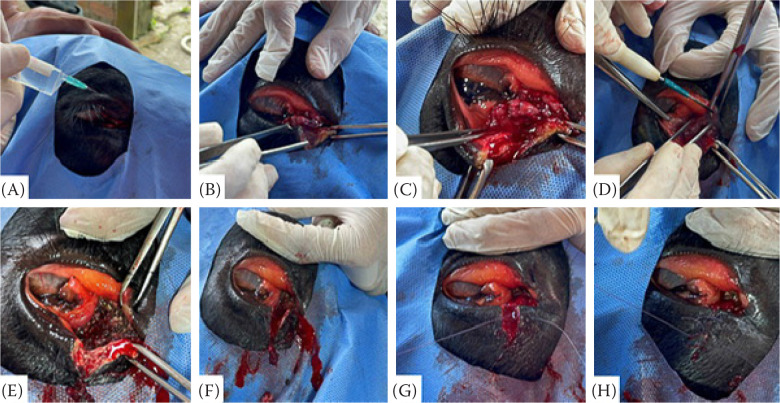
Surgical procedure for excision of squamous cell carcinoma in the right eye (A) Infiltrative anaesthesia in the upper eyelid. (B) Incision of the lower eyelid. (C) Divulsion between the third eyelid tissue and the tumour mass (D). Excision of the tumour mass with an electric scalpel. (E) Removal of the tumour mass. (F) Preservation of the third eyelid and eyeball. (G) Intradermal suture of the proximal layer of the lower eyelid. (H) Simple interrupted suture in the distal portion of the lower eyelid

After antisepsis of the ocular globe with a 0.5% iodine and 0.9% sodium chloride solution, an incision was made in the lower eyelid with a scalpel. The third eyelid was exposed and retracted to excise the tumour mass using electrocautery. Initial suturing was performed intradermally, followed by simple interrupted sutures with absorbable thread (Figure 3). In the immediate postoperative period, a bactericidal and larvicidal repellent (Silverbac^®^ Prata; Labgard Ltda, Porto Alegre, Brazil) was applied around the ocular region. Additionally, 50 mg/kg of dipyrone (Dipirona^®^; IBASA Ltda, Porto Alegre, Brazil) was administered intramuscularly (IM), 5 ml of dexamethasone (Dexaflan^®^; Lema-Injex bioLOGIC Ltda, Minas Gerais, Brazil) IM, and 1 ml/50 kg of ivermectin with abamectin (SOLUTION^®^ 3.5%; MSD Saúde Animal Brasil Ltda, São Paulo, Brazil) subcutaneously (s.c.).

For seven days, 2.5 mg/kg of enrofloxacin (Flobiotic^®^ 10%; Syntec Ltda, São Paulo, Brazil) and 0.6 mg/kg of meloxicam (Meloxinew^®^; Vetnil Ltda, São Paulo, Brazil) were administered i.m., both twice daily (b.i.d.), and an ophthalmic drop containing 0.5% gatifloxacin (Zymar^®^ XD; Allergan Ltda, São Paulo, Brazil) was given three times daily (TID) (Figure 1).

**Figure 4:**
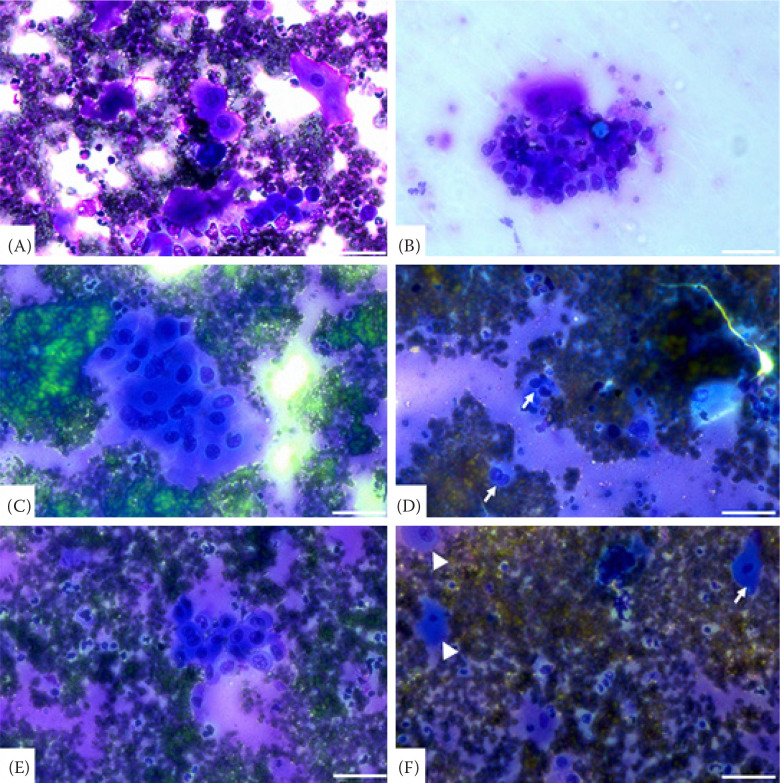
Photomicroscopy of the cytology of the tumorous mass (A) Cellular pleomorphism. (B) Cells in clusters. Round to oval nuclei. (C) Clusters showing pleomorphism with moderate to marked anisocytosis and anisokaryosis. (D) Binucleated cells (arrow). (E) Binucleated cells. Loose chromatin and prominent nucleoli. (F) Large cells with abundant cytoplasm. Marked cellular pleomorphism. The cytoplasm sometimes appears indistinct and is displaced to one side. (“tadpole”) (“arrow”). Moderate to marked anisokaryosis and anisocytosis (arrowhead). Scale bar = 50 μm

During the surgery, four tissue fragments were collected for diagnostic purposes on the day of the procedure (D0). In addition to direct fragment collection, imprint and fine-needle aspiration biopsy (FNAB) were performed for cytological examination.

Throughout the treatment period, physiological parameters (heart rate, respiratory rate, and rectal temperature) remained within the normal physiological range (Table 1). However, the tumour mass demonstrated progressive growth and did not show a significant response to the initial treatments with topical solutions and systemic medications (Figure 2).

**Table 1:** Vital signs measured (mean ± SD) during 4 period of evolution of the ocular tumour in a 10-year-old female buffalo

Vital signs	Days								Normal range (Castro et al. 2020)
	–28 to –21	–21 to –14	–14 to –7	–7 to 0	0 to 7	7 to 14	14 to 21	21 to 28	
Heart rate (beat/minute)	42.2 ± 5.9	48.2 ± 10.3	49.1 ± 7.2	49.7 ± 6.6	41.1 ± 10.1	44.1 ± 7.2	47.4 ± 8.3	35.0 ± 7.6	36–60
Respiratory rate/minute	21.0 ± 4.2	21.7 ± 7.7	25.5 ± 6.9	21.1 ± 3.2	21.8 ± 3.3	25 ± 6.5	27.5 ± 9.9	26.4 ± 4.7	16–30
Temperature (°C)	37.4 ± 0.6	37.8 ± 0.4	38.2 ± 0.6	38.2 ± 0.4	37.8 ± 0.3	38.0 ± 0.3	38.1 ± 0.3	37.6 ± 0.2	37–39

On the day of surgery (D0), the tumour mass was successfully excised while preserving the eyeball and third eyelid. Four tissue samples were collected, with dimensions ranging from 0.9 × 0.9 × 0.6 cm to 1.0 × 1.2 × 0.8 cm. In the immediate postoperative period, analgesics, antibiotics and ophthalmic drops were administered. The initial recovery was considered satisfactory, with no immediate postoperative complications.

Cytological analysis of the collected fragments, performed via imprint and fine-needle aspiration biopsy (FNAB), revealed neoplastic cells suggestive of squamous cell carcinoma, as shown in Figure 4. The cellular features and lack of response to conservative treatment indicated the need for surgical intervention.

**Figure 5:**
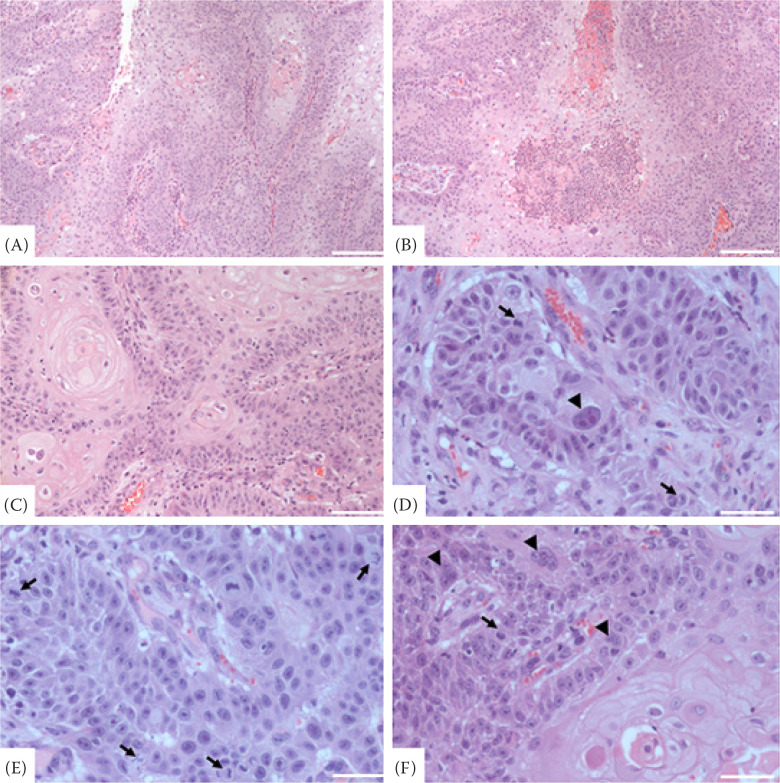
Photomicrography of the histopathology of a nodule in the eyeball of a 10-year-old female buffalo (A,B) Proliferation of squamous epithelial cells with an infiltrative growth pattern and island arrangement. Scale bar = 100 μm. © Islands and trabeculae separated by fibrocollagenous stroma; cellular pleomorphism with moderate to pronounced anisocytosis and anisokaryosis. Scale bar = 50 μm. (D) Round to oval nuclei; prominent karyomegaly (arrowhead); single or multiple conspicuous nucleoli (large arrow); mitoses (small arrow). (E) Mitoses (arrow). (F) Cells with pronounced anisokaryosis; single (arrow), binucleated, and multinucleated nuclei (arrowhead). Scale bar = 20 μm

Histopathological examination revealed proliferation of squamous epithelial cells with an infiltrative growth pattern, organised in islands and trabeculae, and a discrete fibrocollagenous stroma. The cells exhibited moderate pleomorphism, anisocytosis, and anisokaryosis, with eosinophilic cytoplasm and distinct cytoplasmic borders. A total of 43 mitotic figures were observed in 10 high-power fields, some of which were atypical (Figure 5).

**Figure 6:**
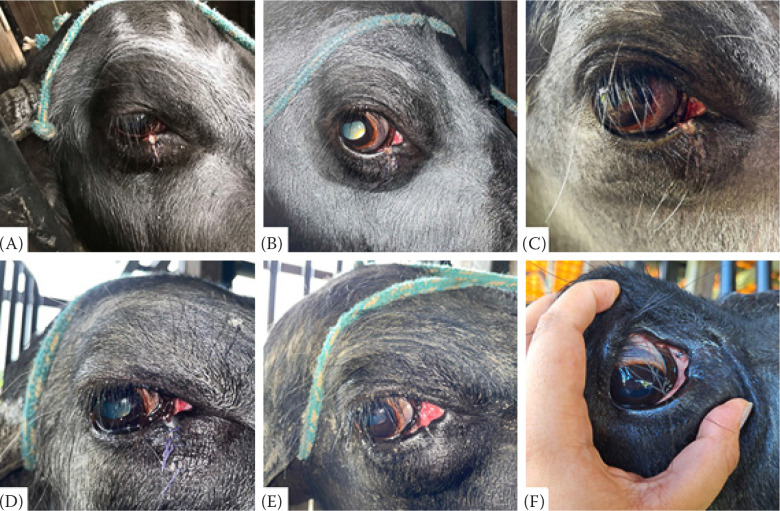
Post-operative recovery after excision of the tumorous mass (A,B) Day 7; (C,D) day 14; (E) day 21 suture removal; (F) day 56

Seven days after surgery (D7), mild ocular discharge was observed, associated with the healing process. The treatment continued with ocular washing using 0.9% saline solution, application of ophthalmic ointment, and use of a repellent spray around the eyes for an additional seven days (D14). After suture removal, during third week (D21), granulation tissue growth was noted, which ceased one week later (D28) and regressed over the next four weeks, leading to complete healing (D56) (Figure 6).

## DISCUSSION AND CONCLUSION

The female buffalo treated for ocular squamous cell carcinoma (OSCC) in this case report was maintained in an extensive farming system, living in open-air pastures with trees providing shade. However, the entire buffalo herd was exposed to sunlight throughout the day. Ultraviolet (UV) rays are a significant carcinogenic factor, as noted by Chahory et al. (2002) and Nithya et al. (2022). Additionally, areas with lower pigmentation and hair loss are more susceptible to tumour development, as melanin provides photoprotection for epidermal and mucosal surfaces (Quevedo et al. 2020). Consequently, the periocular region, the vulvar region (Dariva et al. 2021), the limbs (Regmi et al. 2018), and the ears (Devi et al. 2010) are commonly affected.

In this report, the tumour was detected early and was identified as a cauliflower-shaped, exophytic, friable, hyperaemic, and ulcerative lesion (Krishna et al. 2020). Rapidly growing OSCC, if left untreated, can lead to intracranial invasion or metastasis, as reported by Barros et al. (2006). Surgical intervention was essential to prevent tumour recurrence and progression (Ramos et al. 2007).

Medical support and monitoring were provided to ensure the patient recovered without complications, including secondary infections or pain. Gentle and careful handling is crucial, particularly in neoplasm excisions, due to the sensitivity of the involved areas and their susceptibility to opportunistic bacteria and myiasis (Rizzo et al. 2015). The use of antibiotics, anti-inflammatory drugs, and parenteral analgesics was tailored to the animal’s clinical condition. In this study, the combined use of these treatments led to satisfactory progress, preserving vision and achieving healing without recurrence, consistent with the outcomes reported by Kuma and Sharif (2018) using a similar protocol. Histopathological and cytological examinations confirmed the diagnosis of OSCC, with cellular characteristics consistent with those reported by Srivastav et al. (2022).

Early diagnosis and timely selection of surgical intervention enabled wide-margin excision of the OSCC, successfully preserving ocular anatomy and function postoperatively. This approach resulted in the animal’s satisfactory recovery without complications, underscoring the critical importance of prompt and appropriate treatment in managing such cases.

## References

[R1] Anderson DE, Badzioch M. Association between solar radiation and ocular squamous cell carcinoma in cattle. Am J Vet Res. 1991 May;52(5):784-8. 1854107

[R2] Barros RR, Rech RR, Viott AM, Barros CSL. Carcinoma de celulas escamosas no olho de bovino com invasao cerebral atraves dos nervos cranianos [Ocular squamous cell carcinoma in a cow with cerebral invasion through cranial nerves]. Ciencia Rural. 2006 Sep;36(5):1651-4. Portuguese.

[R3] Carvalho T, Vala H, Pinto C, Pinho M, Peleteiro MC. Immunohistochemical studies of epithelial cell proliferation and p53 mutation in bovine ocular squamous cell carcinoma. Vet Pathol. 2005 Jan;42(1):66-73. 15657274 10.1354/vp.42-1-66

[R4] Castro SRS, Rebelo LS, Junior OSF, Belo-Reis AS, Neves KAL, Silva WC, Morini AC, Vale WG. Influence of seasonality on the physiological and seminal parameters of buffaloes in the western region of Para. Pesqui Vet Bras. 2020 Dec;40(12):1048-53.

[R5] Chahory S, Clerc B, Devauchelle P, Tnibar A. Treatment of a recurrent ocular squamous cell carcinoma in a horse with Iridium-192 implantation. J Equine Vet Sci. 2002 Nov;22(11):503-6.

[R6] Costa RA, Schild C, Caffarena D, Giannitti F, Banchero G, DelpPio L, Riet-Correa F. High frequency of cutaneous squamous cell carcinoma in Friesian Milchschaf sheep in Uruguay. Pesqui Vet Bras. 2019 Apr;39(4):98-109.

[R7] Dariva F, Valduga PC, Lira AL, Colet R, Ruzycki JF, Setim DH, Almeida MA, Backes GT, Oliveira DS. Carcinoma indiferenciado metastatico em bovino leiteiro: Relato de caso [Dairy cattle undifferentiated metastatic carcinoma: A case report]. Rev Persp. 2021 Oct;45(171):15-23. Portuguese.

[R8] Devi VR, Veeraiah G, Annaourna P, Estheru S. Squamous cell carcinoma of ear in an Indian water buffalo (Bubalus bubalis). Braz J Vet Pathol. 2010 Feb;3(1):60-2.

[R9] Dubielzig RR. Tumors of the eye. In: Meuten DJ, editor. Tumors of domestic animals. 4^th^ ed. Iowa: Iowa State Press; 2002. p. 739-54.

[R10] Islam ST, Khurma J, Wani JM, Ganaie MY, Singh AK, Rashid H, Fayaz IB. Ocular squamous cell carcinoma in a female buffalo: A case report. J Entomol Zool Stud. 2017 Nov;5(6):795-6.

[R11] Krishna NVVH, Sreenu M, Nagaraju N. Squamous cell carcinoma of eye in buffaloes – A report of two cases. Buffalo Bull. 2020 Sep;39(3):395-400.

[R12] Kuma KM, Sharif NM. Surgical management of ocular squamous cell carcinoma in buffaloes: A report of 4 cases. J Entomol Zool Stud. 2018 Mar;6(2):402-4.

[R13] Nithya P, Balasubramaniam GA, Arulmozhi A, Gopalakrishnamurthy TR, Kathirvel S. Factors influencing the occurrence of ocular neoplasms in cattle and buffaloes. Pharma Innov. 2022 Dec;11(12):3043-6.

[R14] Oliveira MC. Neoplasias em animais de producao diagnosticadas no Setor de Anatomia Patologica da UFRRJ no período de 1947 a 2019 [Neoplasms in production animals diagnosed in the Department of Pathological Anatomy of UFRRJ from 1947 to 2019] [dissertation]. Seropédica (Brazil): Universidade Federal Rural do Rio de Janeiro; 2020. Portuguese.

[R15] Pires APC. Estudo das enfermidades de animais de producao no estado do Para, diagnosticadas no Setor de Anatomia Patologica da Universidade Federal Rural do Rio de Janeiro, no periodo de 1997 a 2017 [Study of diseases of production animals in the state of Para, diagnosed at the Pathological Anatomy Sector of the Federal Rural University of Rio de Janeiro, from 1997 to 2017] [dissertation]. Seropédica (Brazil): Universidade Federal Rural do Rio de Janeiro; 2018. Portuguese.

[R16] Quevedo DAC, Oliva CAC, Bravo NL, Hernandez DR. Estudio clinico, histopatologico e inmunohistoquimico del carcinoma de celulas escamosas ocular bovino en el departamento de Narino, Colombia [Clinical, histopathological and immunohistochemical study of bovine ocular squamous cell carcinoma in the Narino department, Colombia]. Rev Investig Vet Peru. 2020 Oct;(4):251-4. Spanish.

[R17] Ramos AT, Norte DM, Elias F, Fernandes CG. Carcinoma de celulas escamosas em bovinos, ovinos e equinos: Estudo de 50 casos no sul do Rio Grande do Sul [Squamous cell carcinoma in cattle, sheep and horse: Study of 50 cases in south of Rio Grande do Sul]. Braz J Vet Res Anim Sci. 2007 Jun;44:5-13. Portuguese.

[R18] Regmi S, Regmi S, Lamichhane U, Bista S, Tiwary AK. A case report of squamous cell carcinoma in buffalo. Int J Vet Sci Anim Husb. 2018 Oct;3(6):5-7.

[R19] Reis MO, Slaviero M, Lorenzett MP, Cruz RAS, Guimaraes LLB, Pavarini SP, Driemeier D, Sonne L. Neoplasmas bovinos diagnosticados no Setor de Patologia Veterinaria da UFRGS, Porto Alegre (2005–2014) [Bovine neoplasms diagnosed in the Sector of Veterinary Pathology of UFRGS, Porto Alegre, Brazil (2005–2014)]. Pesqui Vet Bras. 2017 Feb;37(2):105-9. Portuguese.

[R20] Rizzo H, Carvalho JS, Hora JHC, Febronio AMB. Tratamento clinico-cirurgico de carcinoma de celulas escamosas vulvar em bovinos do Estado de Sergipe [Surgical treatment of vulvar squamous cell carcinoma in cattle in the State of Sergipe]. Sci Plena. 2015 Apr;11(4):1-6. Portuguese.

[R21] Santos RL, Alessi AC. Patologia veterinaria [veterinary Pathology]. 2^nd^ ed. São Paulo: Roca; 2016. 856 p. Portuguese.

[R22] Soares MS, Caldeira FHB, Fracasso IO, Araujo KC. Carcinoma de celulas escamosas em conjuntiva ocular de bovino – Relato de caso [Squamous cell carcinoma of the ocular conjunctiva in cattle – Case report]. Rev Ibero-Am Humanid Cienc Educ. 2023 Jan;9(1):428-36. Portuguese.

[R23] Srivastav A, Shyama NP, Singh R, Gangwar NK. Ocular squamous cell carcinoma in a buffalo. J Indian Vet Assoc. 2022 Mar;20(3):112-6.

[R24] Tessele B, Barros CSL. Tumores em bovinos encontrados em abatedouros frigorificos [Tumours found in cattle from slaughterhouse]. Pesqui Vet Bras. 2016 Mar;36(3):145-60. Portuguese.

